# What are the primary care physicians and cardiologists talking about? a cross-sectional analysis of two telemedicine services in Rio de Janeiro, Brazil

**DOI:** 10.1186/s12875-025-02989-6

**Published:** 2025-10-06

**Authors:** Leonardo Graever, Sathya Karunananthan, Rafael Aaron Abitbol, Gabriel Pesce de Castro da Silva, Laís Pimenta Ribeiro dos Santos, Mariana Borges Dias, Marcelo Machado Melo, Viviane Belídio Pinheiro da Fonseca, Leonardo Cançado Monteiro Savassi, Aurora Felice Castro Issa, Anne Frølich, Maria Kátia Gomes, José Roberto Lapa e Silva, Clare Liddy, Helena Dominguez

**Affiliations:** 1 Department of Biomedical Sciences – Faculty of Health and Medical Sciences, University of Copenhagen, Copenhagen, Denmark; 2 Interdisciplinary School of Health Sciences - Faculty of Health Sciences, University of Ottawa, Ottawa, Canada; 3 Secretaria Municipal de Saúde do Rio de Janeiro, Rio de Janeiro, Brazil; 4 Instituto Nacional de Cardiologia, Rio de Janeiro, Brazil; 5 Escola Nacional de Saúde Pública, FIOCRUZ, Rio de Janeiro, Brazil; 6 Ministério da Saúde do Brasil, Brasília, Brazil; 7 Departamento de Medicina de Família e Comunidade, Saúde Mental e Coletiva, Escola de Medicina, Universidade Federal de Ouro Preto, Ouro Preto, Brazil; 8 Department of Public Health, Faculty of Health and Medical Sciences, University of Copenhagen, Copenhagen, Denmark; 9 Departamento de Clínica Médica, Faculdade de Medicina, Universidade Federal do Rio de Janeiro, Rio de Janeiro, Brazil; 10 Department of Family Medicine, Faculty of Medicine, University of Ottawa, Ottawa, Canada; 11 Department of Cardiology, Bispebjerg and Frederiksberg Hospital, Copenhagen, Denmark

**Keywords:** Telemedicine, Primary health care, Cardiovascular disease, Low- and middle-income countries, Continuing medical education

## Abstract

**Background:**

Primary care physicians (PCPs) face challenging clinical situations. Telemedicine between PCPs and specialists involving case discussions in cardiology are frequent. Assessing these interactions is essential for identifying knowledge gaps and tailoring support. In Rio de Janeiro, Brazil, two new telemedicine services provide cardiology support for PCPs: one from the Municipal Health Department using WhatsApp (Meta) and one from the Brazilian Heart Insufficiency with Telemedicine (BRAHIT) research project, which uses a web-based platform. This study analysed and compared the use of these two services in terms of their frequency, distribution among city areas, and content of the PCPs’ questions and cardiologists’ answers.

**Methods:**

Cross-sectional study. We described the demographic characteristics of the patients whose cases were discussed and the primary care physicians’ use frequency. We classified the reasons for encounter and discussed diagnoses using the International Classification of Primary Care (ICPC-3), the question types using the Taxonomy of General Clinical Questions domains, and the specialist’s answers using an adapted version of the Champlain eConsult BASE™ research group’s classification.

**Results:**

We analysed the usage data of all interactions (*N* = 1065) and the detailed content of a random sample (n = 346). The PCPs used the Health Department service more frequently (332/1093, 31%) than the BRAHIT project service (43/1331, 5%). The median answer time was shorter for the Health Department service (19 min) than for the BRAHIT service (two days). Most questions to the health department service were classified within the *diagnosis* domain, mainly regarding electrocardiography interpretation. The questions asked to the BRAHIT service were more frequently classified into *treatment* or *management* domains. The advantages and drawbacks of both models and the contributions of the findings to future implementation projects and continuing medical education opportunities are discussed.

**Conclusions:**

The two types of telemedicine services were adopted differently by the PCPs, with more frequent use and focus on diagnosis in the Health Department WhatsApp (Meta)-based service, compared with less frequent use, more centred on treatment and management topics, in the BRAHIT. Further research using standardised taxonomies for content analysis is needed to inform optimal practices in telemedicine services between providers and guide future initiatives.

## Background

Given the complexity of primary care [1–3], physicians often face uncertainties and dilemmas regarding clinical decisions. For support, they rely on published, organised resources such as books, journals, and online knowledge platforms. Additionally, they frequently engage in *curbside* discussions with colleagues [4, 5] or use telemedicine between providers, where patients’ cases are discussed synchronously or asynchronously, usually involving a primary care physician and their specialised peers [6]. This last strategy, referred as “telemedicine between providers” during this paper to follow the World Health Organisation nomenclature [7], is also known as tele-expertise, teleconsultation or e-consultation between providers [8].

The use of telemedicine between primary care physicians and specialists is not new [9]. There are many large-scale services available globally [10–13], and benefits such as increased primary care physician satisfaction, lower referral rates, and economic savings have been documented in systematic literature reviews [14–18]. Nevertheless, like any innovation, implementing and adopting telemedicine between providers is a complex process, and underuse is frequent [19–22]. The contributing factors include resistance to changes in established practices among professionals, time constraints, unfamiliarity with technology, and a perceived lack of utility. Both human and structural aspects have been extensively studied to inform implementation initiatives and improve the likelihood of success [9, 21, 23, 24].

In Brazil, the national policy on telemedicine was established in 2007 to foster the creation of regional telemedicine nuclei throughout the country [25]. The impact of the policy was heterogeneous among the Brazilian states.

Recently, two telemedicine initiatives were taken to assist primary care physicians in caring for patients with cardiovascular disease in Rio de Janeiro. The BRAHIT telemedicine service was implemented in 2020 within the *Brazilian Heart Insufficiency with Telemedicine (BRAHIT)* project, a binational collaboration among academic institutions from Denmark and Brazil to improve the care of patients with heart failure in Brazil via telemedicine solutions [26]. In 2023, a second telemedicine service was launched by the Sector of Noncommunicable Diseases of the Primary Care Health Department in Rio (hereinafter referred to as the *Health Department telemedicine service*), which uses the WhatsApp (Meta) text messaging platform. Both services worked complementarily to support the decisions of primary care physicians in cardiology.

Studies have shown the importance of assessing telemedicine services from diverse perspectives, considering not only administrative process outcomes such as utilisation and referral avoidance rates and economic savings but also the content of the interactions [27, 28]. The content analysis of the interactions is strategic, providing insights into usability, continued education needs, and implementation aspects of telemedicine between providers. However, reports of the content analysis of these interactions are scarce in the literature [29, 30]. Therefore, this study aims to analyse and compare utilisation rates, the content of questions posed by primary care physicians, and cardiologists’ responses in both services in Rio de Janeiro, Brazil. Our research questions were as follows: How many primary care physicians in Rio de Janeiro have utilised each telemedicine service, which questions have they posed, and which answers have they received? Were there differences between the use of the two services?

## Objectives

Describe primary care physicians’ utilisation of each telemedicine service, concerning frequency, distribution across Rio de Janeiro, and patient characteristics.

Compare and analyse the differences between the two types of telemedicine services, including the reasons for use and the clinical aspects of the interactions between the providers.

## Methods

### Study design

We designed a cross-sectional study to analyse primary care physicians’ use patterns and questions to cardiologists and their answers. We used descriptive statistics to analyse use patterns, the International Classification of Primary Care (ICPC-3) published by the World Association of Family Doctors [31] to classify the reasons for encounters and diagnoses, and the Taxonomy of Generic Clinical Questions (TGCQ) described by Ely et al. [30] to classify the questions. To classify the answers, we adapted the system used by the Champlain eConsult BASE™ service from the University of Ottawa, Ontario, Canada [32–34]. When applicable, we used the *Strengthening the Reporting of Observational Studies in Epidemiology* (STROBE) [35, 36] as a reporting guide.

### Study setting

#### General setting

Brazil is the 5^th^ largest country, with 203 million inhabitants relying on a public, free healthcare system (*Sistema Unico de Saúde – SUS*). Since 2006, upon the publication of the National Policy of Primary Health Care, the country has structured its primary care sector on the basis of the Family Health Strategy [37]. Health professionals are organised in teams comprising one physician, a nurse, a nurse technician, and four to six community health workers. The teams work in primary care practices. Each team covers a delimited geographical area within the practice region and is responsible for primary care delivery for approximately 3000 people. The population also relies on oral health services delivered by dentists and technicians. Other allied professionals compose an extended multiprofessional team, such as psychologists, physical education professionals, and physiotherapists, usually covering a larger area and population than the core primary care teams do [38].

Rio de Janeiro is Brazil's second-largest city, with 6.2 million inhabitants, and the capital of the homonymous State of Rio de Janeiro. The city has 239 primary care practices and 1,358 primary care teams. Healthcare management is divided into ten administrative regions. Each region is led by a regional coordinator and its staff under the guidance of the Health Department. The Municipality Primary Care Department includes technical and administrative staff organised into sections on the basis of groups of health conditions, encompassing noncommunicable diseases [39].

In Brazil, the national telemedicine policy was implemented in 2007, named *Telessaúde Brasil Redes* [25, 40], stimulating the development of state coordination bureaus and at least one telemedicine *nucleus* in each state. There has been notable development of some *nuclei* since then, reaching high teleconsultation numbers and receiving positive feedback from primary care providers in at least three Brazilian states [12, 41, 42]. The Rio de Janeiro *nucleus*, *Telessaúde* RJ, has functioned since 2003, and the only one implemented in Brazil's second most populous state, with over 16 million inhabitants and 3500 primary care teams, does not offer telemedicine between providers, focusing primarily on professional education and, more recently, on telemedicine between providers and patients [43].

#### The BRAHIT project cardiology telemedicine service

The BRAHIT project telemedicine service provided advice on cardiology care from the National Institute of Cardiology of Brazil to primary care physicians in Rio de Janeiro to handle cardiology cases, focused on but not limited to patients with heart failure. The project started in 2019 and was funded by the Danida Fellowship Centre, an organisation from Denmark's Ministry of Foreign Affairs. Since June 2021, the BRAHIT project has provided teleconsultations via an asynchronous web interface to all primary care practices in Rio. Primary care physicians send questions about patients'cases via secure access to the platform and interact with the cardiologist through the platform’s messaging system. A previous paper described the service in detail as the setting of a feasibility study [26]. We analysed data from June 2021 to December 2022. Since 2023, recruitment for a cluster randomised trial assessing the intervention has begun, leading to changes in the inclusion criteria and methodology of the intervention [44]. Therefore, we did not include the BRAHIT project cluster trial participants in this analysis.

#### Health Department’s cardiology telemedicine service

In the Municipality Health Department telemedicine service, one cardiologist, also an author of this paper (RA), answered questions posed by primary care physicians about caring for patients with cardiologic conditions. A WhatsApp (Meta) group was created, and primary care physicians from the city were invited to join. After joining, they could ask written questions through the app answered by the RA. Files and pictures of ECG traces and other image tests could be sent, but audio messages were not allowed. RA answered the questions as soon as they were visualised. Primary care physicians from eight of the ten administrative areas have joined the group. Two administrative areas rely on different support strategies; therefore, the physicians from these areas have not joined the WhatsApp (Meta) group. We analysed the available data collected by the service team from January to December 2024.

### Participants

All primary care physicians who used the Health Department service from January to December 2024 or the BRAHIT project from June 2021 to December 2022 were eligible to participate.

To analyse the diagnoses, the classification of questions, and the answers from the cardiologist in the Health Department service, due to the large number of participants and interactions and the research team’s limited assessment capacity, we used a probability sampling formula to define the sample size, assuming a 95% confidence interval and a 5% error margin for representativeness [45]. We used the RAND() function on Microsoft Excel (Microsoft Corporation) for the random selection. Sample data were checked for representativeness by comparing continuous and categorical variables with the complete dataset. Owing to the low number of entries in the BRAHIT project, sampling in this case was unnecessary, and the whole dataset was included for analysis.

### Data sources and management

Access was granted to the anonymised Health Department telemedicine service database upon request. Records from January to December 2024 were available. From the BRAHIT project, data on all interactions from June 2021 to December 2022 were available on the project’s telemedicine platform, which was managed by the researchers. The following data were extracted from the databases according to their availability (Table 1):

**Table 1:** Collected data and measured variables according to type, source, and method

*Data*	*Type*	*Database*	*Acquisition/calculation method*
Physicians’ data			
Physician’s administrative area of work	Text	BRAHIT/Health Department	Extraction
Number of physicians who used the service	Number	BRAHIT/Health Department	Count
Number of eligible physicians per administrative area	Number	Health Department’s public database	Consultation of public database
Number of interactions with the service per administrative area	Number	BRAHIT/Health Department	Count
Patient’s data			
Age	Number	BRAHIT/Health Department^a^	Extraction
Sex	Text	BRAHIT/Health Department^a^	Extraction
Race	Text	BRAHIT^b^	Extraction
Teleconsultation data			
Date/time of the interaction	Date/time	BRAHIT/Health Department	Extraction
Question content	Text	BRAHIT/Health Department	Extraction
Answer content	Text	BRAHIT/Health Department	Extraction

^a^Extracted from the text of the teleconsultations in the Health Department Service Database, when available

^b^Data from the Health Department Service were not available

When the data were not structured, we extracted information from the text of the teleconsultations, if available (for example, if age or sex was mentioned within a message). We used Microsoft Excel © (Microsoft Corporation) to gather and organise the data stored as local files on the primary author’s computer.

### Data analysis

#### Utilisation data

On the basis of the entire dataset, we calculated the proportion of each service used at least once among PCPs from each administrative area, the medians, and the interquartile ranges for the frequency of interactions per physician/year.

#### Content analysis

We analysed the content of the questions and answers of 278 interactions from the Health Department service and all 68 interactions from the BRAHIT project, totalling 346 questions, for the classification of reasons for encounters and diagnoses, clinical questions, and cardiologists’ answers.

##### Reasons for encounters and diagnoses

For reasons for encounter, we used codes from the *A1* class of the ICPC-3, *visits for general examination and routine examination*, or from component *S, i.e., symptoms, complaints, and abnormal findings*. These codes refer to the reason for the encounter that originated with the teleconsultation interaction. For established diagnoses, we used the codes from component *D—General diagnoses and diseases*. We analysed the frequency of each group separately.

##### Questions

We classified the questions from the PCPs using the Taxonomy of General Clinical Questions (TGCQ) [30], which originally contains four hierarchical levels. The first level comprises six broad categories: *diagnosis, treatment, management, epidemiology, nonclinical questions, and not classified*. Each first-level category is further branched into subcategories, creating secondary, tertiary, and quaternary levels to detail each classification. In total, 64 categories are available and represented by numeric codes. To enhance the classification clarity, we used only the first two levels of this taxonomy system, totalling 26 possible categories. We concluded that using further levels of the taxonomy would not add valuable information and could confound the presentation of the results. The taxonomy levels used are displayed in Table 2.

##### Answers

The answers from the cardiologists were classified using an adapted version of the categories described in the Champlain eConsult BASE™ classification [32], comprising *diagnosis, screening recommendation, investigation recommendation, medications (start/stop/rationale), medications (monitoring/complications), nonpharmacological therapy, complications—comorbidities*, or *other*. We added the categories *clearance for certification*, *referral to outpatient services*, *referral to urgency*, and *multiple recommendations,* the latter used when the answer comprised more than two recommendations. We considered it useful to include those categories owing to particular features of our services and databases. Each assessor could assign two answer categories for each interaction and multiple recommendations when more than two were identified. We measured, for each interaction, whether each answer category was assigned and calculated the proportions of the categories used. The time elapsed between each question and the corresponding answer was calculated on the basis of the date and time records.

**Table 2:** Taxonomy levels used for the classification of the clinical questions (TGCQ) [30]

L	Code	Primary	Secondary
1	1.1	diagnosis	cause/interpretation of clinical finding
2	1.2		criteria/manifestations
3	1.3		test (lab, ECG, imaging, biopsy, skin test, element of physical exam, etc.)
4	1.4		name finding
5	1.5		orientation
6	1.6		inconsistencies
7	1.7		cost
8	1.8		not elsewhere classified
9	2.1	treatment	drug prescribing
10	2.2		not limited to but may include drug prescribing
11	2.3		not elsewhere classified
12	3.1	Management^a^	condition/finding
13	3.2		other providers
14	3.3		doctor‒patient communication
15	3.4		not elsewhere classified
16	4.1	epidemiology	prevalence/incidence
17	4.2		aetiology
18	4.3		course/prognosis
19	4.4		not elsewhere classified
20	5.1	nonclinical	education
21	5.2		administration
22	5.3		ethics
23	5.4		legal
24	5.5		frustration
25	5.6		not elsewhere classified
26	6	unclassified	

Source: Ely JW, Osheroff JA, Gorman PN, Ebell MH, Chambliss ML, Pifer EA, et al. A taxonomy of generic clinical questions: classification study [30]

^a^Not specifying diagnostic or therapeutic

#### Bias reduction efforts

A prior test for the classification of the questions and answers was carried out by the first author (LG), who tested the classification in 20 randomly selected records of teleconsultation interactions of the samples’ spreadsheet, checking for usability and understanding. After a positive evaluation, two other assessors (HD and LCMS) independently classified the same questions. We compared the three assigned values, measuring the proportion of agreement in pairs and among the three authors. We found a mean interrater agreement of 50% when the TGCQ was applied and 83% when the Champlain e-Consult BASE ^TM^ classification was used. The three authors discussed the findings to increase the understanding and agreement of the classification system. This method has been previously used in other studies applying the TGCQ [33, 34], which was originally described as having 55% interrater agreement [30]. Following the discussions, at least one assessor classified all the remaining questions and answers from the sample. For data analysis, we considered the agreed values for the 20 records discussed by the group, and further questions were divided in three equal parts and assessed each by one of the three assessors.

### Statistical analysis

We used descriptive statistics to summarise the data, calculating means, medians, and interquartile ranges for continuous variables and rates, frequencies, and proportions for discrete variables. The statistical software R (R Foundation for Statistical Computing) [46] was used to analyse the data. We managed missing data using appropriate methods to ensure accurate analysis of proportions, medians and means related to demography and text classification. These strategies aimed to minimize the bias of the results, allowing for reliable estimates of demographic data and content analysis despite missing entries.

## Results

### Participants

**Fig. 1:**
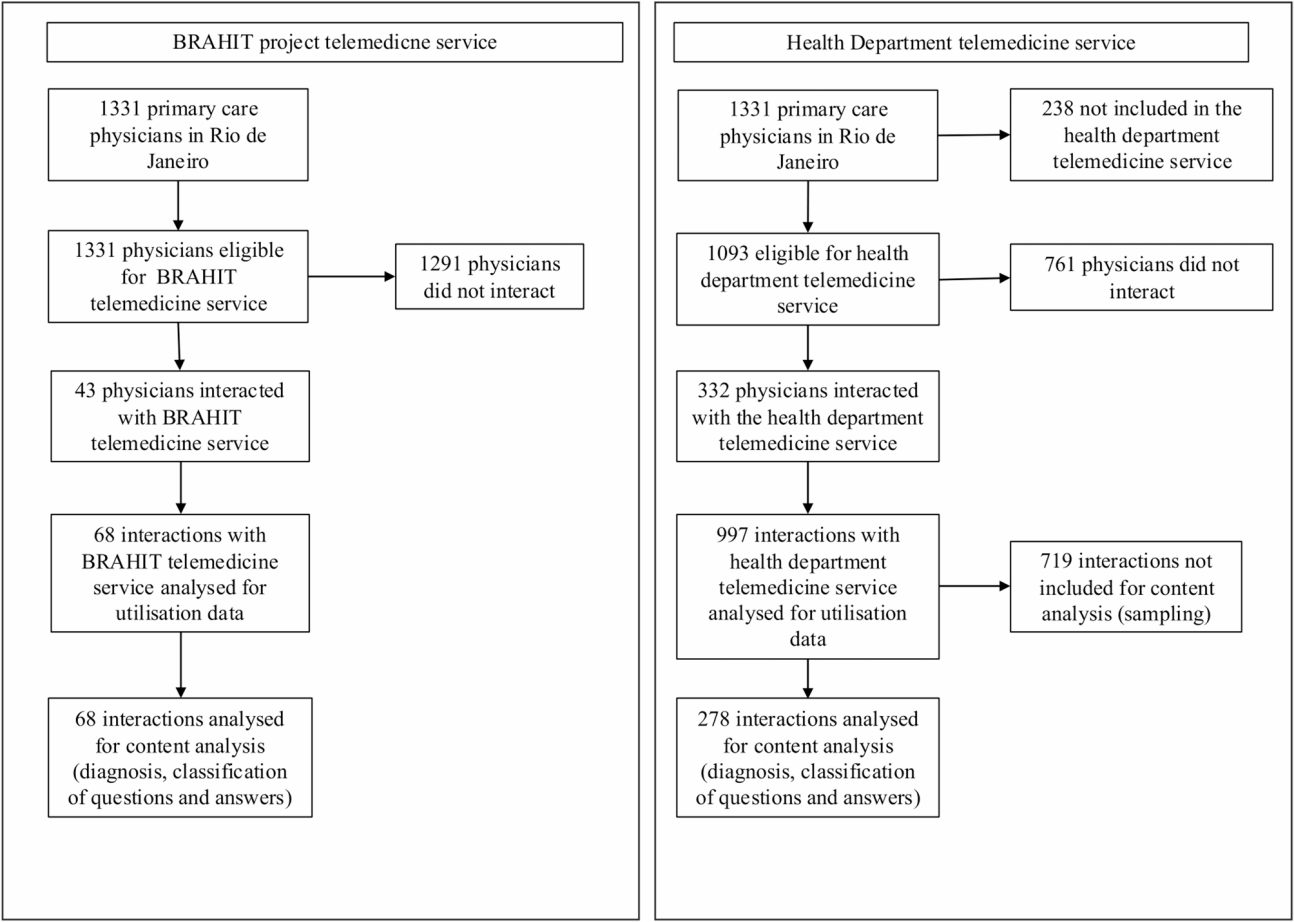
Flowchart of participant inclusion and data analysis according to the telemedicine service

The health department telemedicine service was accessed at least once by 332 of 1093 (31%) physicians to whom the service was offered. The BRAHIT service was used by 43 of the 1331 physicians (5%) to whom the service was offered in 18 months. The inclusion of the participants and analysis flow are systematised in Fig. 1.

### Utilisation data

The median proportion of PCPs that used the service at least once in each administrative area was 25% (IQR 14, 55) for the health department service and 4% (IQR 2,8) for the BRAHIT service. The health department telemedicine service was used 997 times, with a median number of teleconsultations per physician/year of 3.3 (IQR 2.8, 3.7). In contrast, the BRAHIT telemedicine service had 68 interactions, with a median of 0.9 (IQR 0.7, 1.1) interactions per physician/year.

**Fig. 2:**
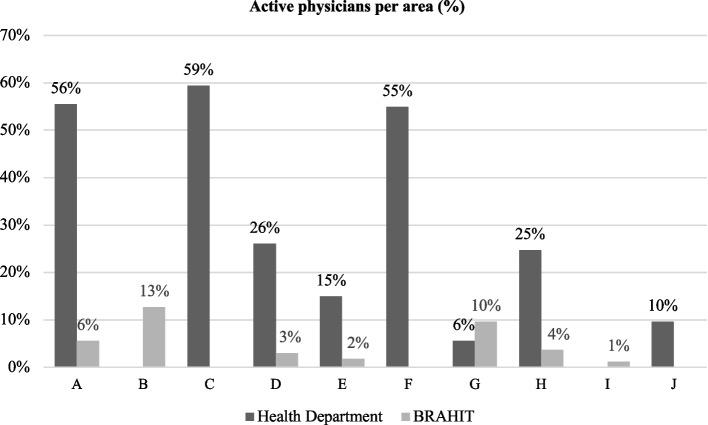
Proportion of physicians who used each telemedicine service per Rio de Janeiro’s Health Department administrative area. The letters A to J are proxies for the real area’s names

**Fig. 3:**
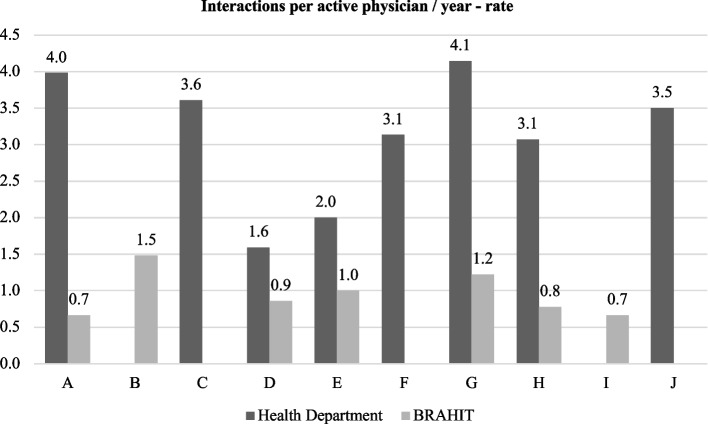
Interactions per physician per year according to the Rio de Janeiro’s Health Department administrative area. The letters A to J are proxies for the real area’s names

Although the use frequency among the administrative areas was variable, no specific distribution pattern was identified. The health department service was not offered in two areas, and there were no interactions with the BRAHIT telemedicine service in three of the ten city areas, although it was offered. We did not include the actual names of the areas, using instead the letters A to J as proxies of the real names. Data concerning the utilisation of each service per area are displayed in Figs. 2 and 3.

There were no discrepancies in age or sex distribution between the services. Race proportions, available only in the BRAHIT service data, reflected the distribution in the research setting, with 23/60 (38%) White and 37/60 (62%) Black or Brown. Table 3 describes the use frequencies and patient demographics.

**Table 3:** Demographic data of patients whose cases were discussed, according to telemedicine service

*Patient data*	*Health Department service*	*BRAHIT service*
*Age*		
Missing, n (%)	283 (26)	0
Median (IQR^a^)	59 (49, 68)	59 (38, 70)
*Sex* n (%)		
Missing	514 (47)	0
Female	245 (51)	29 (43)
Male	238 (49)	39 (57)
*Race, n (%)*		
Missing	N/A	8 (1)
White		23 (38)
Black/Brown		37 (62)

^a^Interquartile range

### Content analysis

For the content analysis of reasons for encounter and diagnoses, questions and answers, we have assessed all the interactions of the BRAHIT telemedicine service and a sample of 278 of the 997 interactions of the Health Department service.

### Reasons for encounters and diagnoses

Analysing the question contents, 585 reasons for encountering and general symptoms or diagnoses were identified and coded from 346 interactions, with an average of 1.7 codes per interaction. We used 234 (40%) codes for reasons for encountering and general symptoms from the ICPC-3 chapter *A1* and component *S—symptoms, complaints, and abnormal findings.* Diagnosis codes from component *D—General diagnoses and diseases* were used 351 times (60%).

The first group of codes was more frequent in the health department service (67/105, 45%) than in the BRAHIT project’s discussions (39/149, 26%). The most frequent reason for an encounter or symptom coded in the health department service was *KS01—pain, pressure, and heart* tightness (67/195, 34%), whereas the most common reason in the BRAHIT service was *RS02—shortness of breath* (24/39, 62%). The code *AG03—Examination and encounter for certification purposes* was frequent in the Health Department service (46/195, 24%), including certification for physical activity (15/195, 8%) or surgery (30/195, 16%), usually involving the interpretation of an ECG.

Regarding diseases and diagnoses, the most frequent code was *KD73—hypertension, uncomplicated* (63/241, 26%) in the health department service and *KD67.01—chronic heart failure* (40/110, 36%) in the BRAHIT project. Other conditions, such as arrhythmias and coronary artery disease, were also frequent in both services. Interactions involving the discussion of at least two comorbidities occurred in 129/256 (50%) in the health department service and 51/68 (75%) in the BRAHIT service. Detailed data on the diagnosis and reasons for encounter can be found in Table 4.

**Table 4:** Diagnoses involved in clinical questions, according to service

Classification (ICPC – code, name). N = 585^a^	Health Department		BRAHIT	
**N, %**	**436**	**%**	**149**	**%**
Symptoms, complaints and abnormal findings	195	45%	39	26%
KS01 Pain, pressure, tightness of heart	67	34%	11	28%
RS02 Shortness of breath	37	19%	24	62%
AG03 Examination and encounter for certification purposes	46	24%	1	3%
AS07 Fainting	13	7%	3	8%
KS02 Palpitations, awareness of heart	13	7%	0	
NS09 Vertigo or dizziness	11	6%	0	
PS01 Feeling anxious or nervous or tense	8	4%	0	
Diagnoses and diseases	241	55%	110	74%
KD73 Hypertension, uncomplicated	63	26%	7	6%
KD67.01 Chronic heart failure	16	7%	40	36%
KD74 Hypertension, complicated	27	11%	15	14%
KD70 Cardiac arrhythmia or conduction disorder or both	31	13%	9	8%
KD66 Chronic ischemic heart disease	23	10%	13	12%
TD72 Type 2 diabetes mellitus	28	12%	3	3%
KD68 Atrial fibrillation or flutter	19	8%	10	9%
ND70 Cerebrovascular disease	8	3%	2	2%
UD66 Chronic kidney disease	4	2%	4	4%
KD71 Heart valve disease	5	2%	2	2%
KD69 Paroxysmal tachycardia	6	2%	1	1%
KD72 Other specified and unknown heart disease	2	1%	4	4%
KD65 Acute coronary syndrome	3	1%	0	
KD99.00 Aortic aneurysm or dissection	2	1%	0	
KD67.03 Left ventricular heart failure with preserved ejection fraction	2	1%	0	
RD68 Chronic obstructive pulmonary disease and emphysema	1	0.4%	0	
KD01 Infection of circulatory system	1	0.4%	0	

^a^Up to three diagnoses were codified per text

### Questions

Primary care physicians accessed the health department service mainly to discuss ECG findings. Therefore, the most frequent first-level classification for the questions was *diagnosis* (203/278, 73%), within which 190 (91%) involved the discussion of an ECG. On the other hand, the BRAHIT project telemedicine service had the most questions classified into *treatment* (30/68, 44.1%) and *management* (29/68, 42.6%) first-level categories.

Among the second-level categories within *treatment*, the most frequent in both services were *drug prescribing,* and *not limited to but may include drug prescribing*. In the *management* classification, interactions with the health department service were associated with medical or administrative doubts about referrals in 13/20 (65%) cases. In comparison, 24/29 (82%) of the questions classified as *management* in the BRAHIT project were associated with managing a specific condition or finding. A detailed overview of the questions’ classification can be seen in Table 5.

**Table 5:** Classification of clinical questions according to service

Question classification, n (%)	Health Department (*n* = 278)	%	BRAHIT (*n* = 68)	%
Diagnosis	203	73%	6	9%
Test (ECG)	190	94%	1	17%
Test (other)	9	4%	4	67%
Diagnosis of a condition or finding	2	1%	1	17%
Not elsewhere classified	1	0.5%	0	0%
Cause/interpretation of clinical finding	1	0.5%	0	0%
Criteria/manifestations	-			
Name finding	-			
Orientation	-			
Inconsistencies	-			
Cost	-			
Not elsewhere classified	-			
Treatment	45	16%	30	44%
Drug prescribing	22	49%	16	53%
Not limited to but may include drug prescribing	22	49%	14	47%
Not elsewhere classified	1	2%	0	0%
Management	20	7%	29	43%
Condition/finding	7	35%	24	83%
Referral to other providers	13	65%	5	17%
Doctor‒patient communication	-			
Not elsewhere classified	-			
Epidemiology	-			
Nonclinical	9	3%	0	0%
Administration	*9*	*100%*	*0*	*0%*
Education	*-*			
Ethics	*-*			
Legal	*-*			
Frustration	*-*			
Unclassified	1	0.4%	3	4%

### Answers

The median answering time in the Health Department telemedicine service was 19 min (IQR 6, 57) and 2.2 days (IQR 0.5, 7) in the BRAHIT project.

The cardiologists’ answers correlated with the needs and inquiries of the primary care physicians. In the health department service, 197 (71%) of the 278 interactions involved the discussion of a diagnosis due to the large proportion of questions about ECG interpretation. The use of the service for orientation about clearance for surgery or physical activity was also evident in 37/278 (13%) of the sample. The BRAHIT project’s service was used mainly to discuss medication prescribing and other clinical management questions. Therefore, the answers were concentrated in the categories of *investigation recommendations* (28/68, 41%) and *medication* (36/68, 53%). The high number of nonclassified answers in the BRAHIT project (26/68, 38%) reflects missing data from telephone or videoconference interactions. The classification of the answers according to each category is available in Table 6.

**Table 6:** Classification of the answers provided by the cardiologists according to the telemedicine service

Classification, n (%)	BRAHIT (*n* = 68)	Health Department (*n* = 278)
Diagnosis	0 (0)	197 (71)
Investigation recommendation	28 (41)	28 (10)
Medications (monitoring/complications)	1 (2)	0 (0)
Medications (start/stop/rationale)	35 (52)	54 (19)
Clearance for certification (surgery, physical activity)	0 (0)	37 (13)
Referral to outpatient services	4 (6)	12 (4)
Referral to urgency services	1 (2)	8 (3)
Non-classified	26 (38)	4 (1)
Nonpharmacological therapy	0 (0)	2 (1)
Multiple recommendations	1 (2)	0 (0)

## Discussion

### Summary of findings

We conducted a cross-sectional analysis of the utilisation frequency, geographical distribution, patient demography, and content of the questions and answers of two telemedicine services in Rio de Janeiro, Brazil. One-third of the city's primary care physicians used the health department service, utilising WhatsApp (Meta) as a communication tool. Most inquiries within the municipal service concerned the diagnosis of ECG tests, drug treatment, and uncertainties regarding referral indications. Requests for clearance for physical activity for healthy individuals and perioperative assessments, typically focused on ECG interpretation, were also common. The service provided timely responses, with an average response time of 50 min, a short response time compared with other e-consultation service standards, which usually aim for a response time between one and seven days [47–49].

The BRAHIT project service utilised a different *modus operandi*. After a teleconsultation request on a web platform, interactions occurred mainly through the platform itself, although in some cases, WhatsApp (Meta), telephone, and videoconference were also used. The average response time was two days. The primary care physician’s level of engagement was low, and interactions focused on treatment, drug prescribing, and the clinical management of complex cases. The signs, symptoms, and diagnoses discussed included the most common reasons for primary care encounters and prevalent diseases in both services, and the responses varied accordingly. Owing to the project's focus on this condition, a higher frequency of heart failure diagnosis was found in the BRAHIT project service.

### Interpretation of findings

#### Use frequency and distribution

Overall, the utilisation rate of both services was low compared with previously established services in the country, which deliver over 22,000 teleconsultations *per* year, even covering less populated states and cities [40, 41]. The fact that those services have been implemented for more than 20 years must be considered in the comparison. However, adoption barriers have been described in our previous research [26] and studies about the hardships of telemedicine between providers’ implementation, which vary depending on the context [21, 24, 50, 51]. They include excessive primary care workload, a lack of users’ perceptions of utility, insufficient training, unfamiliarity with the systems’ technology, the availability of in-person opportunities for case discussion [5, 21, 52], and digital infrastructure, which is most common in low- and middle-income countries [53–55]. Consequently, many projects involving telemedicine between providers do not overcome the pilot phase [56].

The health department service teleconsultations were provided in a timely manner. Although compatible with other services described in the literature, the longer response time of the BRAHIT project (median of 2 days) than that of the health department service (median of 19 min) may have influenced the utilisation rate. Furthermore, the health department service used WhatsApp (Meta), which is a popular communication tool in Brazil. Giordano et al., also Brazilian authors, have conducted a systematic review of WhatsApp (Meta) use in healthcare, describing it as an effective means of communication among professionals [57]. This may explain its higher adoption rate than the BRAHIT project, which relied on a web-based platform. Although they are commonly used for telemedicine between providers’ operations, online platforms may increase workload, especially if not embedded in electronic health records. In a systematic review, workload addition was described as a significant barrier to telemedicine between providers’ utilisation [24].

On the other hand, depending on the completeness of the information exchanged through texting, a complete assessment of the patient’s case may not be feasible, and significant clinical features could be overlooked. For example, a patient with cardiovascular risk factors, typical chest pain, and a normal ECG is a common situation in clinical practice [58]. Depending on the primary care physician's familiarity with chest pain guidelines, the advice should extend beyond ECG interpretation and diagnosis. Providing further and thorough recommendations based on good clinical practice guidelines can improve the quality of the service and the patients’ safety in these cases [59], even if not requested by the primary care physician.

Another possible reason for the lower use of the BRAHIT service is the connection to a research project. The literature describes a low adherence of primary care physicians in local research projects [60, 61]. Moreover, the service was not an official offering from the city’s health department.

#### Diagnosis, questions, and answer classification

Our findings enabled us to identify the most common reasons for the use of telemedicine between providers by primary care physicians in Rio de Janeiro for supporting the management of cardiovascular conditions. Analysing the content of questions and answers in telemedicine services between providers is crucial for thoroughly evaluating primary care physicians’ drive for using the service, the main discussion topics, possible knowledge gaps, and the input of specialists to primary care physicians, providing valuable feedback for quality improvement.

The results were consistent with the typical frequency observed in primary care [62] regarding reasons for encounters and diagnoses. Multimorbidity was common, with over half of the interactions involving multiple diagnoses, highlighting the complexity of medical practice in primary care and underscoring the necessity for collaborative support from other healthcare sectors [63, 64].

The taxonomy developed by Ely et al. and the Champlain eConsult BASE ™ classification were extremely useful in mapping physicians’ questions and cardiologists’ answers, respectively. In another study using the same systems, Karunananthan et al. discussed the importance of using taxonomic classifications of clinical questions in telemedicine between providers, highlighting the need for more extensive studies in different settings [32].

The most frequent reason for questions in the health department service was the interpretation of an ECG, which remains a challenge for primary care physicians in this research context and for non-cardiology health professionals in general [65]. In a study on telemedicine between providers in cardiology conducted at the Veterans Affairs medical centres in New England [13], 92.7% of teleconsultations between primary care physicians and cardiologists addressed clinical problems, and the two most common types of questions were related to interpreting test results or defining the most appropriate therapy, similar to our findings.

In the same study, 7.3% of requests were related to administrative matters. In our study, 6.9% of the requests (24 out of 346) involved either administrative issues or ECG interpretations for physical activity, often in children, which can also be regarded as administrative, since they typically stem from requests by third parties rather than investigation decisions made by primary care physicians. In a cohort study by Ahmed et al. [27], the appropriateness of teleconsultation requests, defined as the suitability of the question for telemedicine use, was analysed using a framework developed by the authors. One of the four criteria was whether the request sought administrative information, leading to an inefficient use of specialised resources. In addition, one must also consider the work and financial burden on primary care services caused by unnecessary tests, particularly when the evidence supporting the need for medical clearance is weak [66].

In the BRAHIT telemedicine service, questions regarding administrative and diagnostic interactions were less frequent, leading to more discussions about treatment and management. These findings align with results from a study on real-time responses by librarians conducted by Bjerre et al. [67], where treatment, encompassing drug selection and prescribing practices, was the most common category of interaction. The fact that options for prompt discussions, such as ECG diagnosis, for example, were not available could also have influenced this proportion.

### Strengths and limitations

Our study has several strengths. This was the first study to assess two different telemedicine models between providers in a low- or middle-income country using standard, previously reported classifications. The analysis of two distinct services allowed us to evaluate the benefits and drawbacks of each strategy, which can inform future telemedicine service implementations. Our method is reproducible because we used validated or publicly accessible classification systems. The large number of interactions enhanced the validity of our findings. The study was pragmatic because it included assessing a service based on WhatsApp (Meta), a widely used communication tool in the study setting.

Our study has several limitations. The first was the discrepant participant numbers (43 *versus* 332) and different time assessments (18 *versus* 12 months) between the BRAHIT and the Health Department telemedicine services, respectively. Our analysis was limited by the low frequency of use of the BRAHIT service and our capacity to assess all 997 participant interactions in the Health Department Service, which led us to select a random sample representative of the interactions (278, 28%) in the latter. Therefore, our data must be considered as descriptive and exploratory, for we could not use inferential statistics to calculate statistical significance or power when comparing the two services. Nevertheless, our observations have shown tendencies and patterns deemed useful and meaningful for the researchers, as described and interpreted above.

Second, our study is observational. Evaluating effectiveness, superiority, or impact on relevant outcomes requires stricter methodologies. We have published a feasibility study [26] and are undertaking a clinical trial in the same setting to achieve these aims [44].

Third, missing data was frequent. In the Health Department service, race data were unavailable, and age and sex data were missing in 26% and 47% of the records, respectively. In the BRAHIT service, 26 out of 68 (38%) answers could not be assessed due to a lack of registration on the web platform. Additionally, we were unable to access the entire content of occasional interactions via other tools, such as WhatsApp (Meta) texting, videoconferencing, or phone calls, in the BRAHIT project. Although we know that these interactions occurred in a few cases, we acknowledge that missing data may have limited the accuracy of our data analysis. We rigorously monitor data collection in our ongoing cluster randomisation trial to minimise similar limitations [44].

Fourth, although we assume that the recommendations from cardiologists to PCPs were, in principle, accepted, our data collection did not include following up on patients or reviewing electronic health records to determine whether they were implemented.

Despite its limitations, our study offers local insight into the implementation of telemedicine services between providers with diverse operational approaches*.*

### Implications and recommendations

Different offers can fit different purposes for diverse audiences in medical education, including the delivery and use of e-health technologies. The health department service’s primary strength lies in its accessibility, facilitating instant interactions and prompt responses, which benefits primary care physicians, who often face time constraints.

Embedding telemedicine between providers’ systems within electronic health records is the gold standard for implementing telemedicine between providers. It facilitates engagement, allows adequate interaction registration, and supports legal procedures and liability via a balanced approach between parallel, time-consuming platforms on one side and informal communication on the other [68].

Our study highlights the importance of analysing the content of questions using a systematic and validated approach. This process allows comparisons between studies, provides valuable feedback for continuing education initiatives [67, 69], and may serve as a foundation for digital solutions and the implementation of large language-learning models utilising artificial intelligence. Consequently, this analysis can be strategic in the research and accountability of telemedicine services between providers [50, 53].

Another recommendation for the next steps in accountability and research in telemedicine between providers is the assessment of the clinical and operational outcomes of the discussions. While agreements related to emergency services or outpatient clinic referrals were derived from texts or databases, we cannot confirm that those outcomes occurred. Measuring the effectiveness of telemedicine between providers can be challenging, as strong data management and accountability culture within the service is necessary.

## Conclusion

The implementation of telemedicine between providers is challenging. Two telemedicine services were adopted differently by the PCPs, with a higher use rate and diagnosis focus in one service, compared with a lower utilisation rate, which was more centred in treatment and clinical management in the other. Studying utilisation patterns and clinical content is paramount for evaluating telemedicine services in clinical research and implementation science. Our study shows that different telemedicine service models may generate diverse usage patterns and interaction content. Our findings suggest that a trade-off between prompt communication and structured delivery may be key for achieving high-quality, scalable telemedicine services between providers. This analysis is valuable for informing managers, medical education stakeholders, and technology developers about providers’ telemedicine needs, ultimately improving the quality of these systems.

## Data Availability

The datasets generated and analysed during the current study are not publicly available owing to the sensitive content regarding clinical discussions between physicians but are available, anonymised, from the corresponding author upon reasonable request.

## Abbreviations

BASE^TM^: Building Access to Specialists through eConsultation (trade mark)
BRAHIT: Brazilian Heart Insufficiency with Telemedicine
ECG: Electrocardiogram
ICPC-3: International Classification of Primary Care – 3
IQR: Interquartile Range
PCPs: Primary Care Physicians
STROBE: Strengthening the Reporting of Observational Studies in Epidemiology
SUS: Sistema Único de Saúde (Unified Health System)
TGCQ: Taxonomy of generic clinical questions
